# Vitamin C Inhibits Triple-Negative Breast Cancer Metastasis by Affecting the Expression of YAP1 and Synaptopodin 2

**DOI:** 10.3390/nu11122997

**Published:** 2019-12-06

**Authors:** Liping Gan, Vladimir Camarena, Sushmita Mustafi, Gaofeng Wang

**Affiliations:** 1 John P. Hussman Institute for Human Genomics, Dr. John T. Macdonald Foundation Department of Human Genetics, University of Miami Miller School of Medicine, Miami, FL 33136, USAvcamarena@med.miami.edu (V.C.); sxm1241@med.miami.edu (S.M.); 2 State Key Laboratory of Animal Nutrition, College of Animal Science and Technology, China Agricultural University, Beijing 100193, China; 3 Sylvester Comprehensive Cancer Center, University of Miami Miller School of Medicine, Miami, FL 33136, USA

**Keywords:** vitamin C, triple-negative breast cancer, metastasis, HIF-1α, YAP1, synaptopodin 2, lamellipodia, F-actin, hippo pathway

## Abstract

Vitamin C supplementation has been shown to decrease triple-negative breast cancer (TNBC) metastasis. However, the molecular mechanism whereby vitamin C inhibits metastasis remains elusive. It has been postulated that vitamin C reduces the levels of HIF-1α, the master regulator of metastasis, by promoting its hydroxylation and degradation. Here, we show that vitamin C at 100 µM, a concentration achievable in the plasma in vivo by oral administration, blocks TNBC cell migration and invasion in vitro. The protein level of HIF-1α remains largely unchanged in cultured TNBC cells and xenografts, partially due to its upregulated transcription by vitamin C, suggesting that HIF-1α unlikely mediates the action of vitamin C on metastasis. Vitamin C treatment upregulates the expression of synaptopodin 2 and downregulates the expression of the transcription coactivator *YAP1*, both genes in the Hippo pathway. The changes in *SYNPO2* and *YAP1* expression were subsequently validated at mRNA and protein levels in cultured TNBC cells and xenografts. Further experiments showed that vitamin C treatment inhibits F-actin assembly and lamellipodia formation, which correlates with the changes in *SYNPO2* and *YAP1* expression. Overall, these results suggest that vitamin C inhibits TNBC metastasis by affecting the expression of *SYNPO2* and *YAP1*. Vitamin C may thus have a potential role in the prevention and treatment of TNBC metastasis.

## 1. Introduction

Metastatic invasion to organs such as bone marrow and lung is the major cause of death for breast cancer patients. Hypoxia-inducible factor 1α (HIF-1α) is the master regulator of cancer cell metastasis [1]. The hydroxylation of HIF-1α, which is a key step toward its degradation, requires vitamin C as a cofactor for HIF prolyl-hydroxylase [2]. Humans cannot synthesize vitamin C de novo due to a mutant and nonfunctional L-gulonolactone oxidase (*Gulo*), the enzyme catalyzing the final step of vitamin C biosynthesis [3]. Vitamin C is thus an essential micronutrient for humans and needs to be consumed by a vitamin C-rich diet or supplements. Vitamin C enters breast epithelial cells mainly through sodium-dependent vitamin C transporters (SVCT). However, the expression of sodium-dependent vitamin C transporter 2 (SVCT2) is decreased in breast cancer cells compared to normal breast epithelial cells from the same patients [4]. Lower SVCT2 expression is shown to cause intracellular vitamin C deficiency in cancer cells [5], suggesting that there is likely intracellular vitamin C deficiency in breast cancer. Taken together, there is a possibility that in breast cancer, lack of vitamin C hinders the degradation of HIF-1α, which in turn promotes metastasis. Thus, supplementation of vitamin C could help decrease HIF-1α levels in breast cancer, which may be beneficial for blocking metastasis.

Although studies on vitamin C and breast cancer metastasis in human patients are still lacking, in vivo animal models confirm the inhibition of metastasis by vitamin C supplementation. For example, oral vitamin C supplementation blocks murine breast cancer metastasis in *Gulo* knockout mice, which like humans, cannot synthesize vitamin C [6]. High doses of vitamin C via intraperitoneal injection inhibit metastasis of human breast cancer xenografts in nude mice [7], which, like the *Gulo* wild-type mice, maintain endogenous vitamin C at ~50 μM in the plasma [8]. Our earlier work also showed that oral vitamin C supplementation blocks human triple-negative breast cancer (TNBC) xenograft metastasis in mice [9]. Due to the lack of targeted therapy, TNBC is usually associated with a more aggressive clinical course and poor survival [10]. Vitamin C, a readily available micronutrient, has been shown to be beneficial in TNBC treatment by inhibiting metastasis in animal models. However, whether HIF-1α is involved in this action of vitamin C remains unclear.

The pharmacokinetics of vitamin C shows a unique, nonlinear relationship between doses and levels in the blood [11]. The average concentration of vitamin C in the plasma of healthy humans is ~50 μM, similar to the levels in *Gulo* wild-type mice. Intravenous infusion of high doses of vitamin C can increase the plasma concentration to mM levels, which quickly drops to ~200 μM. Oral delivery of vitamin C can maintain plasma vitamin C at ~100 μM, which cannot be further elevated by higher doses. Elevating vitamin C by oral delivery, intravenous infusion, or intraperitoneal injection could in principle help compensate for the downregulated SVCT2 expression and increase the uptake of vitamin C by breast cancer cells.

In addition to promoting the degradation of HIF-1α, vitamin C has a previously unrecognized function in epigenetic regulation, identified and verified by different groups, specifically enhancing the demethylation of DNA and histones [12]. Furthermore, our previous study showed that vitamin C suppresses histone acetylation (H3ac and H4ac) in TNBC cells mainly by upregulating the expression of histone deacetylase 1 (HDAC1) [9]. Vitamin C thus is poised to affect the transcriptome of TNBC cells via HIF-1α, DNA demethylation, histone deacetylation, and potentially histone demethylation and other secondary mechanisms. In an attempt to understand the molecular mechanism by which vitamin C inhibits TNBC metastasis, we examined the expression of HIF-1α and other potential candidate genes in cultured TNBC cells and xenografts. Contrary to our initial reasoning, the results showed that inhibition of TNBC metastasis by vitamin C is largely independent of HIF-1α.

## 2. Materials and Methods

### 2.1. Cell Culture and Treatment

MDA-MB-231 and BT-549 TNBC cell lines were purchased from ATCC and maintained under a 5% CO_2_ atmosphere in RPMI medium (Lonza, Walkersville, MD, USA) with 10% heat-inactivated fetal bovine serum (FBS), 100 Units/mL of penicillin, and 100 μg/mL of streptomycin. The cells were incubated for 24 h after seeding and subsequently treated with vitamin C (sodium ascorbate, Sigma-Aldrich, St. Louis, MO, USA). The medium was changed daily to ensure the presence of fresh vitamin C.

### 2.2. Cell Invasion Assay and Migration Assay

A cell invasion assay was performed using the CytoSelect-24 well cell assay (Cell Biolabs, San Diego, CA, USA). Briefly, the cells were pretreated with 0, 50, or 100 µM vitamin C for 2 days. After pretreatment, 2.5 × 10^4^ cells were transferred into an invasion chamber containing membrane inserts with 8 µm pores coated with extracellular matrix (protein matrix isolated from tumor cells, collagen, and laminin). The cells were allowed to invade across the membrane for 48 h and then stained with crystal violet. Five random fields of view (cells that penetrated the membrane) were counted per well. Data are presented as mean ± SEM. A scratch assay was conducted to evaluate cell migratory ability. Briefly, 1 × 10^5^ cells were plated into 6-well plates and pretreated for 5 days with 50 µM or 100 µM vitamin C. Linear wounds were created in cell monolayers using a sterile pipette tip. Images were captured and documented at 0.5 or 20 hours (h) using an Olympus 1 × 50 inverted microscope with a 10× objective lens. Eight images per treatment were analyzed to determine the average position of the migrating cells at the edges of the scratch. Closure was determined by calculating the percentage of distance that cells had migrated.

### 2.3. Immunoblot

MDA-MB-231 cells were seeded into 6-well plates and treated for 5 days with either 0 or 100 μM vitamin C. Cell lysates were collected in radioimmunoprecipitation assay (RIPA) buffer (Thermo Scientific, Waltham, MA, USA) containing a protease inhibiter cocktail (Sigma-Aldrich, St. Louis, MO, USA), 1% SDS, and 0.5 mM dithiothreitol (DTT). The protein concentration was determined by the BCA protein assay (Thermo Scientific, Waltham, MA, USA). Cell lysates were resolved by SDS-PAGE, transferred to polyvinylidene fluoride (PVDF) membranes (Bio-Rad Laboratories, Hercules, CA, USA), and immunoblotted with anti-HIF-1α antibody, anti-YAP1 antibody, anti-cyclophilin B (PPIB) antibody and, anti-β-actin antibody (Cell Signaling Technology, Danvers, MA, USA). Proteins were visualized by using chemiluminescence ECL (Thermo Scientific, Waltham, MA, USA). Specific band densities were quantified by using ImageJ and analyzed by Student’s *t*-test, at α = 0.05.

### 2.4. Immunofluorescence

MDA-MB-231 cells were pretreated with either 0 or 100 µM vitamin C for 3 days in 10 cm dishes before splitting and seeding on coverslips in a 24-well plate. Vitamin C treatment was continued for two additional days. The cells were then washed with cold PBS and fixed for 30 min at room temperature with 4% paraformaldehyde, permeabilized with 0.4% Triton X-100 for 20 min, and blocked with 3% BSA at room temperature for 30 min. The cells were incubated with primary antibodies overnight at 4 °C. After washing with PBS, the cells were incubated with secondary antibodies (Alexa Fluor 488-conjugated donkey anti-rabbit) and 4′,6-diamidino-2-phenylindole (DAPI) for 1 h at room temperature. The cells where washed 3 times with PBS, and the coverslips were mounted with mowiol 4-88 mounting medium (Polysciences, Warrington, PA, USA). The primary antibodies used include anti-SYNPO2 (#HPA030665, Sigma-Aldrich, St. Louis, MO, USA), anti-HIF-1alpha, and anti-YAP1 (#3716S, and #14074; Cell Signaling, Danvers, MA, USA). CF640R phalloidin (Biotium, Fremont, CA, USA) was used to visualize F-actin. All fluorescence images were acquired using a Zeiss LSM 710 confocal microscope and captured into a 1024 × 1024 or 2048 × 2048 frame size by averaging 4 times at a bit depth of 16. The intensities of the images were quantified using ImageJ. The average intensity of each cell within the image field was measured from a minimum of five 20× images per condition. The intensity values of individual cells were plotted and statistically analyzed by Student’s *t*-test, at α = 0.05. The lamellipodia extent was quantified as described previously [13]. Briefly, F-actin was stained with phalloidin, and the flattened F-actin-rich leading edge of the cell was outlined and measured. The summed length of the lamellipodia was normalized to the total cell circumference. Quantification was done using the average of 50 cells per condition.

### 2.5. Xenograft Immunohistochemistry

Xenografts of MDA-MB-231 were collected from previous published experiments [9]. The tumors were fixed in 4% paraformaldehyde overnight at room temperature, and subsequently, 6 μm paraffin-embedded sections were used for staining. For histological examination, the sections were stained with hematoxylin and eosin (H&E). For immunohistochemistry, the above described primary antibodies for immunofluorescence were used. After incubation with biotinylated secondary antibodies and horse radish peroxidase (Vectastain^®^ Elite ABC Reagent, Vector, Peterborough, UK), diaminobenzidine (Vector ImmPACT DAB substrate, Vector, Peterborough, UK) was applied for 5 min before mounting in Permount (Fisher Scientific, Hampton, NH, USA). The sections were subsequently imaged using a Nikon eclipse 50i microscope and a Qimaging micropublisher 5.0 rtv camera.

### 2.6. Gene Silencing

SiRNA against human TET1, TET2, and TET3 were purchased from Dharmacon (Lafayettte, CO, USA) and transfected following the manufacturer’s instruction. Briefly, MDA-MB-231 cells were plated and grown until achieving ~50% confluence. Transfection of siRNA was performed using Lipofectamine 2000 (Thermo Fisher, Waltham, MA, USA). The medium was changed after 6 h of transfection to eliminate the possible toxicity of the transfecting reagents. The cells were then used for invasion assays or harvested after 3 days for RNA extraction.

### 2.7. Quantitative Real-Time PCR

Total RNA was extracted from TNBC cells using RNeasy kits (Qiagen). A nanodrop 8000 photospectrometer was used to quantify the RNA (Thermo Scientific, Waltham, MA, USA). The qScript Flex cDNA kit (Quanta Biosciences, Beverly, MA, USA) was used for reverse transcription, according to the manufacturer’s instructions. Quantitative real-time RT-PCR (qRT-PCR) was performed in triplicate on a Quantstudio 12K flex system (Thermo Scientific, Waltham, MA, USA), using the PowerUp SYBR green master mix (Thermo Scientific, Waltham, MA, USA). Primers were designed to span introns (*SDHA* forward 5′-GCCAGGGAAGACTACAAGGTGCG-3′, reverse 5′-GAATGGCTGGCGGGACGGTG-3′; *HIF-1α* forward 5′-GGTTCACTTTTTCAAGCAGTAGG-3′, reverse 5′-GTGGTAATCCACTTTCATCCATT-3′; *SYNPO2* forward 5′-CTCGCCCCTGTCAAGACTG-3′, reverse 5′-CCAGGCTGTACCGCTTCTA-3′; *YAP1* forward 5′-GAACTCGGCTTCAGGTCCTC-3′, reverse 5′-GGTTCATGGCAAAACGAGGG-3′). The transcript amplification results were analyzed with the QuantStudio 12K Flex software, and all values were normalized to the levels of the SDHA gene using the 2^−(ΔΔCt)^ method. Statistical significance of differences in expression levels was assessed by Student’s *t*-test, at α = 0.05.

### 2.8. Actin Segmentation

The actin segmentation analysis was done using a method described previously [14]. Briefly, cells were lysed directly using actin stabilization buffer (50 mM PIPES (pH 6.9), 50 mM NaCl, 5 mM MgCl_2_, 5 mM EGTA, 2 mM ATP, 5% glycerol, 0.1% Nonidet P-40, 0.1% Triton X-100, 0.1% Tween 20, 0.1% β-mercaptoethanol, 1:100 protease inhibitor mixture, and 1:100 phosphatase inhibitor mixture (Sigma-Aldrich, St. Louis, MO, USA). The cells were collected into tubes, homogenized with a 28G syringe, and incubated at 37 °C for 10 min, followed by centrifugation at 300 × *g* at room temperature to remove insoluble particles. The lysate was centrifuged at 100,000 × *g* at 37 °C for 1 h to precipitate F-actin and to separate the G-actin that remained soluble in the supernatant. The pellet containing F-actin was resuspended and dissociated with 10 µM cytochalasin D (Sigma-Aldrich, St. Louis, MO, USA). Both fractions were resolved by SDS-PAGE and probed with an anti-β-actin antibody. The ratio of F-actin over G-actin was determined and analyzed by Student’s *t*-test.

### 2.9. Statistical Analysis

All data were normalized to those of inner controls, such as housekeeping peptidylpropyl isomerase B (PPIB) expression level. Data are presented as mean ± standard error of the mean (SEM.). Statistically significant changes amongst treatments were assessed by Student *t* tests at α = 0.05.

## 3. Results

### 3.1. Vitamin C Inhibits the Invasion of TNBC Cells

**Figure 1:**
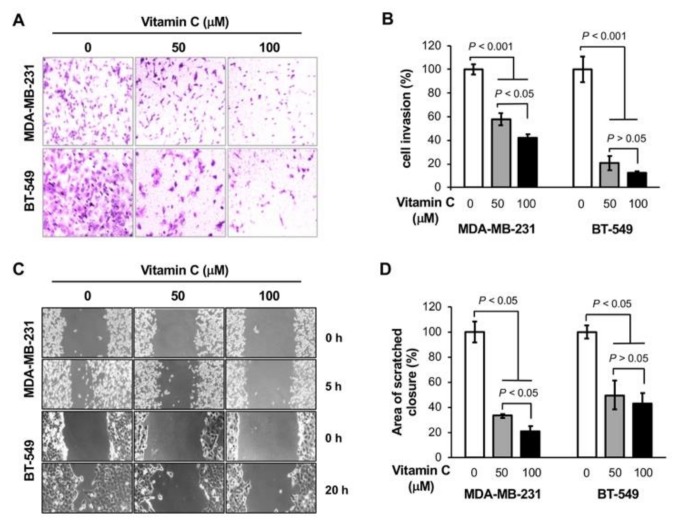
Vitamin C blocks the invasion and migration of triple-negative breast cancer (TNBC) cells. (**A**,**B**) Representative images and quantification show that vitamin C treatment inhibits the invasion of MDA-MB-231 and BT549 cells; (**C**,**D**) representative images and quantification show that vitamin C treatment delays the migration of MDA-MB-231 and BT-549 cells.

Our earlier work showed that oral vitamin C supplementation alone did not affect the growth of MDA-MB-231 xenografts but significantly inhibited metastasis to the liver in NOD severe combined immune deficiency (scid) gamma (NSG) mice [9]. To verify the effect of vitamin C on TNBC metastasis, we conducted an in vitro invasion assay. MDA-MB-231 and BT-549 TNBC cells were pretreated with vitamin C (0, 50, 100 µM) for 3 days and then seeded into invasion chambers. In the presence of different doses of vitamin C, the cells were allowed to invade for two additional days. The number of invading cells were significantly decreased (*p* < 0.001) for both MDA-MB-231 and BT-549 cells treated with vitamin C, as shown by imaging (Figure 1A) and quantification (Figure 1B). Vitamin C at 100 µM further decreased the number of MDA-MB-231 invading cells compared to 50 µM. To test if TET-mediated DNA demethylation is involved in the effect of vitamin C on cell invasion, TETs were silenced in MDA-MB-231 cells by siRNA. Vitamin C only moderately inhibited the invasion of cells transfected with TETs siRNA, while it markedly blocked the invasion of cells transfected with scramble siRNA (Figure S1). These results suggest that TETs could partially mediate the effect of vitamin C on TNBC metastasis.

To further validate the effect of vitamin C on TNBC metastasis, a scratch assay was performed. As shown in Figure 1C,D, vitamin C treatment inhibited the migration of both MDA-MB-231 and BT-549 cells after scratching (*p* < 0.05). Vitamin C at 100 µM further blocked migration of MDA-MB-231 cells compared to the concentration of 50 µM. A cell survival assay was then performed to examine if the effect of vitamin C on invasion and migration was influenced by cell growth. Treatment with vitamin C (50 or 100 µM) did not significantly change the growth of the two TNBC cell lines (Figure S2). Overall, these results suggest that vitamin C at 100 µM, a concentration achievable in the plasma in vivo by oral supplementation, blocks the migration and invasion of TNBC cells in vitro, confirming the inhibitory effect of oral vitamin C on TNBC metastasis in vivo [6,9].

### 3.2. The Level of HIF-1α Protein is Not Altered in TNBC Cells by Vitamin C Treatment

**Figure 2:**
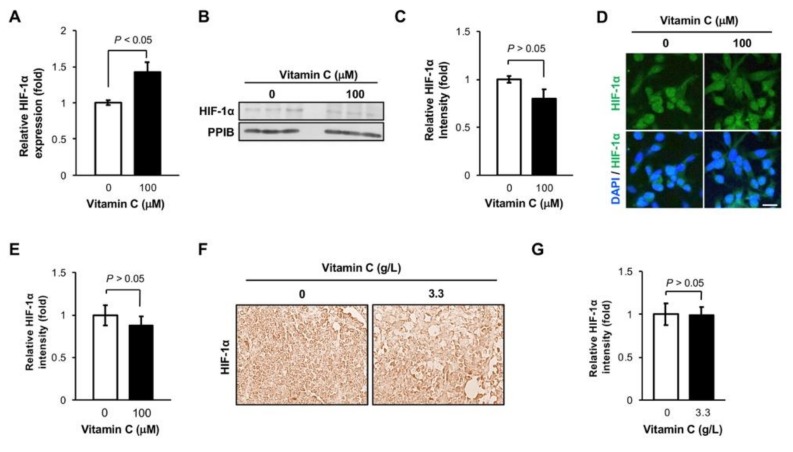
Hypoxia-inducible factor 1α (HIF-1α) protein level remains largely unchanged after vitamin C treatment. (**A**) qRT-PCR shows that HIF-1α RNA is increased in MDA-MB-231 cells after vitamin C treatment for 5 days; (**B**,**C**) HIF-1α protein level remains unchanged in MDA-MB-231 cells after vitamin C treatment, as shown by immunoblotting and semi-quantification; (**D**,**E**) no obvious changes are found in HIF-1α immunofluorescence in MDA-MB-231 cells after vitamin C treatment, as shown by imaging and semi-quantification. Bar = 20 µm; (**F**,**G**) immunostaining (40×) and semi-quantification show no differences in HIF-1α expression in MDA-MB-231 xenografts from NOD combined immune deficiency (scid) gamma (NSG) mice administered or not vitamin C (3.3 g/L) in the drinking water.

HIF-1α is the master regulator of metastasis, and vitamin C promotes HIF-1α degradation [2], suggesting that vitamin C could inhibit metastasis by decreasing HIF-1α protein level. Even under normal cell culture condition (5% CO_2_ atmosphere), the transcripts of HIF-1α were relatively highly expressed in MDA-MB-231 cells, and their expression increased by ~1.5-fold after vitamin C (100 µM) treatment for 3 days on the basis of our published RNA-seq data [4]. Subsequently qRT-PCR showed that HIF-1α RNA level was indeed increased in MDA-MB-231 cells by ~1.5-fold after treatment with 100 μM vitamin C for 5 days (*p* < 0.05, Figure 2A). However, no obvious changes were detected in HIF-1α protein level after this latter vitamin C treatment, as shown by immunoblotting (Figure 2B,C) and immunofluorescence (Figure 2D,E). The housekeeper protein PPIB was used as a loading control. We then examined MDA-MB-231 xenografts collected in our previous study, which showed that oral vitamin C supplementation alone inhibited metastasis to the liver of mice [9]. We found that HIF-1α immunostaining remained at a similar level in MDA-MB-231 xenografts collected from mice supplemented with or without vitamin C (3.3 g/L in the drinking water, Figure 2F,G). These results suggest that vitamin C supplementation does not alter the level of HIF-1α protein in TNBC cells, which may be explained by the counteractions of an increased transcription and enhanced degradation by vitamin C.

### 3.3. Vitamin C Increases Synaptopodin 2 Expression in TNBC Cells

**Figure 3:**
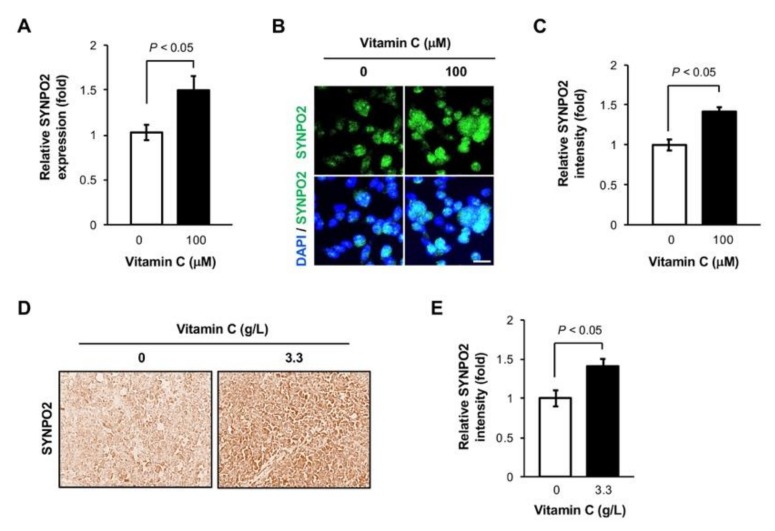
Vitamin C upregulates the expression of synaptopodin (*SYNPO2*). (**A**) *SYNPO2* RNA is increased in MDA-MB-231 cells after treatment with vitamin C for 5 days, as shown by qRT-PCR; (**B**,**C**) immunofluorescence and semi-quantification show increased *SYNPO2* protein level in MDA-MB-231 cells after vitamin C treatment. Bar = 20 µm; (**D**,**E**) the level of *SYNPO2* protein is higher in MDA-MB-231 xenografts from NSG mice supplemented with vitamin C (3.3 g/L) compared to mice without supplementation, as shown by immunostaining (40×) and semi-quantification.

No obvious changes in HIF-1α protein level after vitamin C treatment indicated that other genes, at least within the transcriptome, could underpin the observed reduced TNBC metastasis. By reviewing the known genes involved in breast cancer metastasis [15,16,17] and our available MDA-MB-231 cell RNA-seq data [4], we identified synaptopodin 2 (*SYNPO2*), with a known function in metastasis, which showed the highest fold change (2.05-fold increase) in MDA-MB-231 cells after vitamin C treatment. Studies have shown that breast cancer has a reduced amount of *SYNPO2*, which is associated with higher TNBC metastasis and lower patient survival [18]. Using qRT-PCR, we found that the RNA level of *SYNPO2* was elevated in MDA-MB-231 cells after vitamin C (100 µM) treatment for 5 days (*p* < 0.05, Figure 3A). The upregulated expression of *SYNPO2* after this vitamin C treatment was then verified at the protein level by immunofluorescence (*p* < 0.05, Figure 3B,C). Furthermore, *SYNPO2* expression was also higher in MDA-MB-231 xenografts from mice administered vitamin C (3.3 g/L) compared to mice receiving water without vitamin C (Figure 3D,E). Collectively, these results suggest that vitamin C upregulates the expression of *SYNPO2*, which is known to inhibit TNBC metastasis.

### 3.4. Vitamin C Decreases YAP1 Expression in TNBC Cells

**Figure 4:**
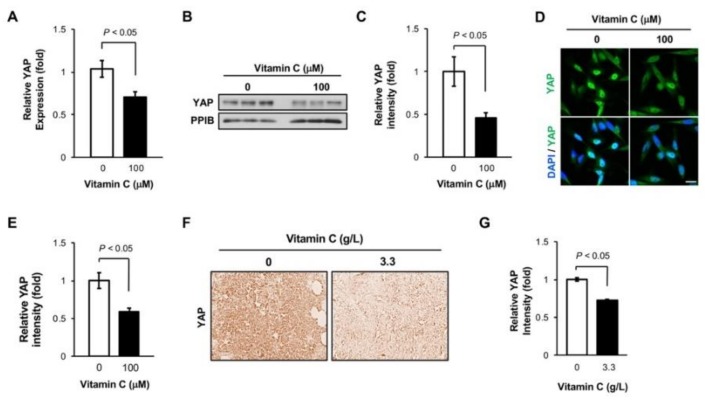
Vitamin C downregulates yes-associated protein 1 (*YAP1*) expression. (**A**) q-RT-PCR shows that *YAP1* RNA level is decreased in MDA-MB-231 cells after treatment with vitamin C; (**B**,**C**) immunoblot and semi-quantification show decreased *YAP1* protein in MDA-MB-231 cells after vitamin C treatment; (**D**,**E**) vitamin C treatment reduces *YAP1* immunofluorescence signal in MDA-MB-231 cells, as shown by imaging and semi-quantification. Bar = 20 µm; (**F**,**G**) The level of *YAP1* protein is lower in MDA-MB-231 xenografts from NSG mice supplemented with vitamin C (3.3 g/L) compared to mice without supplementation, as shown by immunostaining (40×) and semi-quantification.

*SYNPO2* has been found to suppress metastasis by inhibiting the activity of the transcriptional coactivator *YAP1*, also known as yes-associated protein 1 [19]. As a key regulator in the Hippo pathway, *YAP1* promotes focal adhesion formation and metastasis [20]. By inquiring our RNA-seq data, we found that *YAP1* was downregulated in MDA-MB-231 cells by vitamin C (100 μM) treatment. qRT-PCR validated the downregulation of *YAP1* in MDA-MB-231 cells after treatment with vitamin C (100 µM) for 5 days (*p* < 0.05, Figure 4A). Subsequently, immunoblotting and immunofluorescence analyses showed that the protein level of *YAP1* was also reduced in MDA-MB-231 cells after the same treatment (*p* < 0.05, Figure 4B–E). It is known that *YAP1* shuttles into the nucleus and promotes the transcription of genes responsible for metastasis [21]. We then evaluated the subcellular location of *YAP1* by comparing its immunofluorescence signal in the nucleus with the its overall signal. The results showed that vitamin C treatment decreased the ratio of *YAP1* in the nucleus, suggesting the inhibition of *YAP1* nuclear translocation (Figure S3). Further experiments confirmed that there was a significant *YAP1* expression decrease in MDA-MB-231 xenografts from mice supplemented with vitamin C (Figure 4F,G). Taken together, these results suggest that vitamin C treatment inhibits the expression of *YAP1*, which may underpin at least partially the observed reduced TNBC metastasis.

### 3.5. Vitamin C Reduces Lamellipodia in MDA-MB-231 Cells

**Figure 5:**
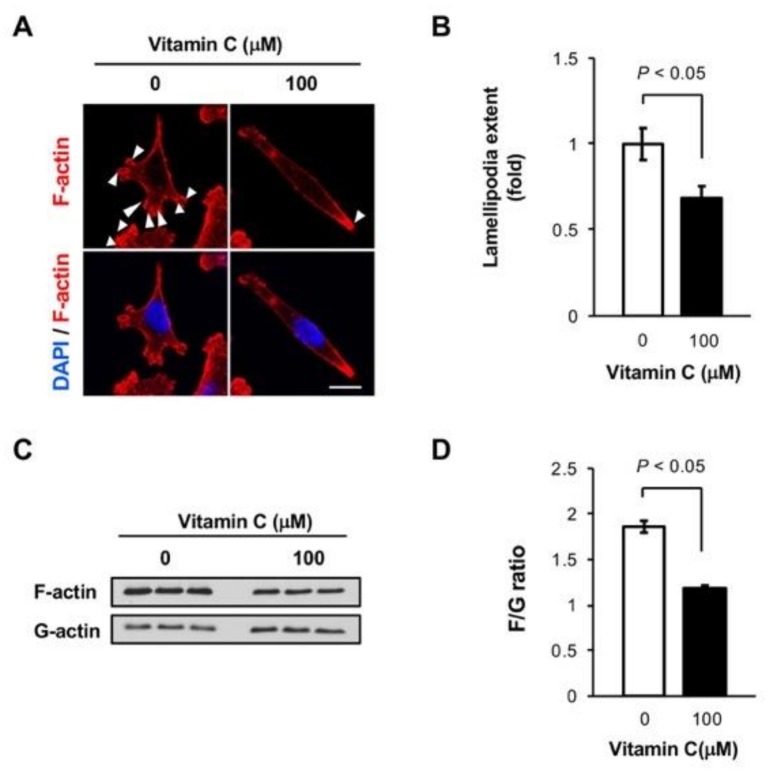
Vitamin C inhibits the formation of F-actin and lamellipodia. (**A**,**B**) Vitamin C treatment reduces F-actin assembly, labeled by phalloidin, and lamellipodia (arrow heads) in MDA-MB-231 cells, as shown by imaging and semi-quantification; (**C**,**D**) the F-actin/G-actin ratio is decreased after vitamin C treatment for 5 days, as shown by immunoblot of fractionated MDA-MB-231 cell samples and semi-quantification.

*YAP1* promotes metastasis by regulating actin dynamics [14], which involves the assembly of filamentous actin (F-actin) from monomeric actin (G-actin) to form lamellipodia to the advantage of cell motility [22,23]. To understand how vitamin C blocks metastasis, we first examined the impact of vitamin C on F-actin and lamellipodia, which were visualized by phalloidin labeling. After vitamin C treatment, we observed an obvious reduction in lamellipodia compared with non-treated cells (Figure 5A,B). We then evaluated the ratio of F-actin over G-actin to examine if vitamin C changed the assembly of actin. There was a significant reduction of the F-actin/G-actin ratio after vitamin C treatment (Figure 5C,D). These results indicate that vitamin C treatment inhibits the formation of F-actin and lamellipodia, which is correlated with the upregulation of *SYNPO2* and the downregulation of *YAP1*. These events could underlie at least partially the action of vitamin C on TNBC cell invasion in vitro and xenograft metastasis in vivo.

## 4. Discussion

Inadequate vitamin C intake has been associated with the risk of breast cancer and the mortality of breast cancer patients. For instance, a meta-analysis of 17,696 patients showed a statistically significant association between the use of vitamin C supplements and reduced mortality [24]. The downregulated expression of SVCT2 in breast cancer could underpin the requirement of additional vitamin C supplementation in breast cancer patients [4]. While vitamin C can be administered orally or by intravenous infusion or intraperitoneal injection, oral vitamin C is easily accessible and more convenient for patients. Possibly due to the difficulty to control vitamin C consumption quantitatively in human subjects, the benefit of oral vitamin C against breast cancer metastasis has remained unclear in patient care. TNBC is often associated with early metastasis and short overall survival [10]. In animal models, oral vitamin C supplementation has been shown to inhibit TNBC metastasis to other organs [6,9], indicating the benefit of oral vitamin C for TNBC metastasis inhibition and calling for further examination of the molecular mechanisms by which vitamin C blocks metastasis.

Intratumoral hypoxia, a common condition in cancer, triggers the expression of HIF-1α which in turn initiates the progression of breast cancer toward metastasis [25]. HIF-1α is thus a prime candidate to mediate the inhibition of TNBC metastasis by vitamin C, because of its known role in HIF-1α hydroxylation and further degradation [2]. However, our results showed that vitamin C does not change the overall HIF-1α protein level. This likely results from the combined effects of enhanced degradation of HIF-1α and upregulated transcription of HIF-1α by vitamin C treatment. It was shown that the transcription of HIF-1α is enhanced by KDM4C-mediated histone demethylation, such as of H3K9me3 and H3K9me2, as well as by TET-1-mediated DNA demethylation [26,27], both of which can be promoted by vitamin C, which thus serves as a cofactor for these iron- and 2-oxoglutarate-dependent demethylases [12]. Therefore, vitamin C increases HIF-1α transcription on one hand and augments HIF-1α protein degradation on the other hand, which results in a largely unaltered HIF-1α protein level, as we discovered in cultured TNBC cells and xenografts. Overall, HIF-1α unlikely mediates the role of vitamin C in blocking TNBC metastasis.

Oral vitamin C administration can easily maintain the plasma level of vitamin C at 100 µM, a concentration which markedly inhibited TNBC xenograft metastasis in vivo [6,9] and TNBC cell invasion in vitro, as shown above. Loss of 5-hydroxymethylcytosine (5hmC) is an epigenetic hallmark of breast cancer and other cancers [28]. Treatment with vitamin C at 100 µM restored 5hmC content in TNBC cells toward the level of non-cancerous breast epithelial cells and shifted the transcriptome [4]. We found that after silencing TETs, vitamin C less effectively inhibited TNBC invasion, suggesting that TETs-mediated DNA demethylation is likely involved in the action of vitamin C on TNBC metastasis. In addition to TET-mediated DNA demethylation, vitamin C can modulate transcription in TNBC cells by enhancing histone deacetylation, as shown in our earlier work [9], and potentially histone demethylation as well. Some of the genes whose expression was altered by vitamin C could in principle underpin the action of vitamin C on metastasis. In this study, we examined *SYNPO2,* which whose expression in MDA-MB-231 cells was most significantly changed by vitamin C treatment, and subsequently its related partner *YAP1*. Both proteins are key players in the Hippo pathway and have known functions in metastasis formation, consistently with the changes we observed in cultured TNBC cells and xenografts after vitamin C treatment. Activation of *YAP1* promotes F-actin assembly and lamellipodia formation, which are essential to cell mobility and invasion [19]. Our results show that vitamin C treatment downregulates the expression of *YAP1* and upregulates that of *SYNPO2*, which further inhibits the activity of *YAP1*. Subsequently, vitamin C treatment inhibits both F-actin assembly and lamellipodia formation, which correlates with the changes in *YAP1* and *SYNPO2* and likely underpin the effect of vitamin C, at least partially, on TNBC invasion and metastasis.

## 5. Conclusions

In summary, vitamin C inhibits TNBC metastasis independently of HIF-1α and, likely, by affecting the expression of *YAP1* and *SYNPO2*, two genes in the Hippo pathway which regulate cell mobility and cancer metastasis. These results suggest a potential role of oral vitamin C supplementation in the prevention and treatment of TNBC metastasis.

## Supplementary Materials

The following are available online at https://www.mdpi.com/2072-6643/11/12/2997/s1, Figure S1: The inhibition of TNBC cell invasion by vitamin C is partially abolished by TETs siRNA; Figure S2: Vitamin C (100 µM) does not affect TNBC cell proliferation; Figure S3: Vitamin C increases YAP1 nuclear translocation.
