# Supplementary material for: Vitamin C Inhibits Triple-Negative Breast Cancer Metastasis by Affecting the Expression of YAP1 and Synaptopodin 2
Source: Nutrients. 2019 Dec 6;11(12):2997. doi: 10.3390/nu11122997 (PMC6950019; doi:10.3390/nu11122997)
Supplement: Supplementary file 1 [file nutrients-11-02997-s001.pdf]

Supplementary Materials

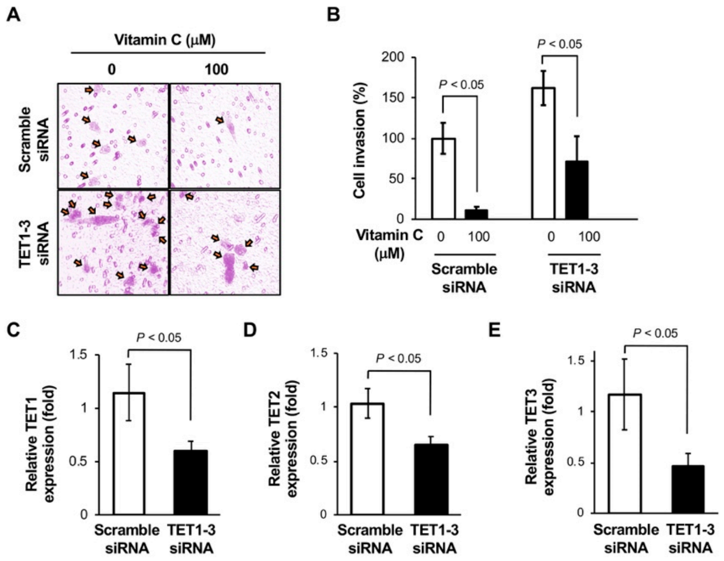

Figure S1. The inhibition of TNBC cell invasion by vitamin C is partially abolished by TETs siRNA. (A-B) Representative images and quantification show that vitamin C treatment inhibits the invasion of MDA-MB-231 transfected with either TETs siRNA or scramble siRNA as controls. However, vitamin C only moderately inhibits the invasion of the cells transfected with TETs siRNA while markedly blocks the invasion of the cells transfected with scramble siRNA. (C-E) The mRNA levels of TET1-3 is reduced by siRNA transfection in MDA-MB-231 cells shown by qRT-PCR.

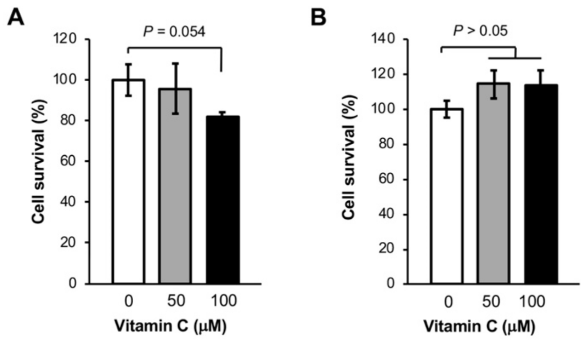

Figure S2. Vitamin C (100 μM) does not affect TNBC cell proliferation. (A-B) The survival rate is not significantly changed in MDA-MB-231 by treatment with vitamin C (50, 100 μM) for 5 days. (B) The survival rate of BT-549 cells is not significantly changed by treatment with vitamin C (50, 100 μM) for 5 days.

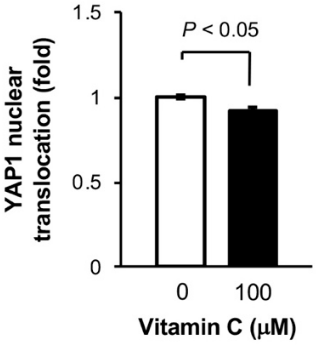

Figure S3. Vitamin C increases YAP1 nuclear translocation. After treatment with vitamin C (100 μM) for 5 days, the presence of nuclear YAP1 show a moderate but statistically significant decrease ($P < 0.05$).
